# Visuomotor dysconnectivity as a candidate mechanism of psychomotor agitation in major depression

**DOI:** 10.1017/S0033291725102638

**Published:** 2025-11-28

**Authors:** Victor Joseph Pokorny, Zachary Anderson, Allison M. Letkiewicz, Dusan Hirjak, Sebastian Walther, Stewart Shankman, Vijay Mittal

**Affiliations:** 1 Department of Psychology, Northwestern University, Evanston, IL, USA; 2 Stahl Center for Psychiatric Neuroscience, Department of Psychiatry and Behavioral Sciences, Northwestern University, Evanston, IL, USA; 3 Department of Psychiatry and Psychotherapy, Central Institute of Mental Health, Medical Faculty Mannheim, University of Heidelberg, Mannheim, Germany; 4 German Centre for Mental Health (DZPG), partner site Mannheim-Heidelberg-Ulm, Mannheim, Germany; 5 University of Bern, Translational Research Center, University Hospital of Psychiatry and Psychotherapy, Bern, Switzerland; 6 University of Würzburg, Department of Psychiatry, Psychosomatics, and Psychotherapy, Würzburg, Germany; 7 Medical Social Sciences, Northwestern University, 625 N Michigan Ave, Floor 21, Chicago, IL, 60611, USA; 8 Institute for Policy Research (IPR), Northwestern University, 2040 Sheridan Rd, Evanston, IL, 60208, USA; 9 Institute for Innovations in Developmental Sciences (DevSci), Northwestern University, 625 N Michigan Ave, Floor 24, Chicago, IL, 60611, USA; 10 Institute for Adolescent Mental Health and Well-Being, Northwestern University, 2029 Sheridan Road, Evanston, IL, 60208, USA

**Keywords:** force variability, psychomotor disturbance, resting state fMRI

## Abstract

**Background:**

Psychomotor disturbance has long been observed in major depressive disorder (MDD) and is thought to be a key indicator of illness course. However, dominant methods of measuring psychomotor disturbance, via self-report and clinician ratings, often lack objectivity and may be less sensitive to subtle psychomotor disturbances. Furthermore, the neural mechanisms of psychomotor disturbance in MDD remain unclear.

**Methods:**

To address these gaps, we measured psychomotor agitation via a force variability paradigm and collected resting fMRI in 47 individuals with current MDD (cMDD) and 93 individuals with remitted MDD (rMDD). We then characterized whether resting-state cortico-cortical and cortico-subcortical connectivity related to force variability and depressive symptoms.

**Results:**

Behaviorally, individuals with cMDD exhibited greater force variability than rMDD individuals (*t*(138) = 3.01, *p* = 0.003, Cohen’s *d* = 0.25). Furthermore, greater force variability was associated with less visuomotor connectivity (*r*(130) = −0.23, *p* = 0.009, 95% CI [−0.38, −0.06]). Visuomotor connectivity was significantly reduced in cMDD relative to rMDD (*t*(130) = −2.77, *p* = 0.006, Cohen’s *d* = −0.24) and mediated the group difference in force variability (ACME β = −0.06, 95% CI [−0.16, −0.01], *p* = 0.04).

**Conclusions:**

Our findings represent a crucial step toward clarifying the pathophysiology of psychomotor agitation in MDD. Specifically, altered visuomotor functional connectivity emerged as a candidate neural mechanism, highlighting a promising direction for future research on dysfunctional visually guided movements in MDD.

## Introduction

Reports of psychomotor disturbance in major depressive disorder (MDD) extend back thousands of years to the writing of Hippocrates and Aretaeus of Cappadocia (Buyukdura, McClintock, & Croarkin, 2011; Sobin & Sackeim, 1997). Despite such long-standing observations, relatively little is known about the etiology of psychomotor disturbance in depression. In modern psychiatric research, psychomotor disturbance is often disaggregated into two dimensions: psychomotor slowing and agitation. Typical manifestations of psychomotor slowing include slowed speech, response times, and gait. Psychomotor agitation, on the other hand, often manifests as restlessness, fidgeting, muscle tension, and irritability. Importantly, psychomotor slowing and agitation can co-occur in the same individual such that agitation and slowing may reflect distinct manifestations of depression rather than two poles of a unitary spectrum (Leventhal, Pettit, & Lewinsohn, 2008).

Psychomotor agitation and slowing are thought to be critical markers of illness severity and treatment responsiveness (Ulbricht, Dumenci, Rothschild, & Lapane, 2016, 2018; van Diermen et al., 2019; Walther, Bernard, Mittal, & Shankman, 2019). For example, Ulbricht et al. (2018) found that men with MDD and psychomotor agitation were less likely to achieve symptom resolution when treated with citalopram compared to other depressive subtypes. Thus, certain treatments may be less effective for individuals presenting with psychomotor disturbance. Clarifying the provenance of psychomotor disturbances may lead to a better understanding of the heterogeneity within MDD and lead to interventions that are better tailored to the individual (i.e., precision psychiatry).

Previous studies of psychomotor disturbance in MDD are often hampered by imprecise measurement, often relying on self-report and/or clinician ratings of psychomotor disturbance (Gorwood, Richard-Devantoy, Baylé, & Cléry-Melin, 2014; Parker et al., 1993; Parker & McCraw, 2017; Song et al., 2024; Ulbricht et al., 2016, 2018). While these methods are valuable, they may be less sensitive to subtle manifestations of psychomotor disturbances (Lohr, May, & Caligiuri, 2013; Razavi et al., 2011; van Diermen et al., 2018). For example, Razavi et al. (2011) demonstrated that expert ratings of agitation were inconsistently correlated with actigraphy based activity levels. Furthermore, clinician ratings require extensive training, which severely limits the scalability and accessibility of psychomotor assessment.

To overcome the aforementioned limitations, the present work examines a potential instrumental measure of psychomotor agitation: variability of force exerted when pressing the index finger on a sensor (i.e. force variability). Force variability increases as a function of age (Potvin et al., 1980) and is altered in individuals with tardive dyskinesia (Caligiuri & Lohr, 1989; Vrtunski, Alphs, & Meltzer, 1991), neuroleptic-naive schizophrenia (Caligiuri & Lohr, 1994), bipolar disorder (Lohr & Caligiuri, 2006), and posttraumatic stress disorder (Keller-Ross et al., 2014). To our knowledge, only one extant study has assessed force variability in the context of MDD (Lohr et al., 2013). In this study, Lohr et al. (2013) found that unmedicated MDD patients showed increased force variability relative to healthy control subjects.

The present work seeks to extend previous work by examining the neurobiological mechanisms of increased force variability in MDD. Lohr et al. (2013) hypothesized that increased force variability may be mediated subcortically via the basal ganglia. Consistent with this, Wüthrich et al. (2024) observed altered resting state connectivity between the primary motor cortex (M1) and putamen in agitated MDD based on clinical ratings of psychomotor agitation. For this reason, we examined whether force variability was related to resting state connectivity between M1 and striatal regions, specifically the caudate and putamen. The cerebellum is also hypothesized to be an important driver of psychomotor dysfunction (Mittal, Bernard, & Walther, 2021). Consistent with this, Wüthrich et al. (2024) observed altered connectivity between lobules 4 and 5 of the cerebellum and M1 in depressed patients with clinician-rated psychomotor agitation. Thus, in the present study, we assessed whether altered connectivity between M1 and lobules 4 and 5 of the cerebellum was associated with force variability. However, as argued by others (Northoff et al., 2021; Song et al., 2024), psychomotor disturbance is likely more globally mediated than previously thought, with sensory networks and the visual cortex specifically seeming to be implicated. Moreover, because the force variability task requires coordination of visual and motor signals to adjust force based on real-time visual feedback, we hypothesized that connectivity between the visual and motor cortex may be a possible mechanism of increased force variability. Finally, the degree to which psychomotor agitation is present in individuals with remitted MDD (rMDD) is an open question. Some work suggests that psychomotor slowing is scar-like: remitted individuals retain some degree of slowing even during symptom remission (Gorwood et al., 2014; Wüthrich et al., 2022). However, less is known about whether a similar pattern is observed for instrumentally measured psychomotor agitation. Thus, by comparing cMDD with rMDD, we sought to clarify whether psychomotor agitation differs across illness phases. We predicted that individuals with cMDD would exhibit more force variability and dysconnectivity between the motor hand area and the following regions of interest: caudate, putamen, cerebellum, and early visual cortex.

## Subjects and methods

Participants were recruited using online and public transportation advertisements, flyers, and community health referrals. Exclusion criteria included head injury, neurological disorder, tic disorder, lifetime ADHD, current alcohol/substance use disorder, and use of psychotropic medications that impact motor function, such as bupropion, antipsychotics, or regular use of benzodiazepines. The primary inclusion criteria for this study was current or remitted MDD using the Structured Clinical Interview for DSM-5 (First, Williams, Karg, & Spitzer, 2015). Participants were considered remitted if they met criteria for past but not current MDD. The Inventory of Depression and Anxiety Symptoms (IDAS) was the primary measure of current depressive symptom severity (Watson et al., 2007). We also obtained clinical ratings of psychomotor retardation and agitation using the CORE (Parker & McCraw, 2017). The authors assert that all procedures contributing to this work comply with the ethical standards of the relevant national and institutional committees on human experimentation and with the Helsinki Declaration of 1975, as revised in 2008.

As depicted in Table 1, groups did not significantly differ on age, sex, race, ethnicity, or education variables. Both groups were predominantly female and white, although the sample had a fair amount of racial and ethnic diversity. Groups did not significantly differ with respect to medication usage; antidepressant usage was almost identical between groups. As expected, the currently depressed group exhibited higher average IDAS depression symptoms and greater CORE psychomotor agitation ratings. Groups did not differ in terms of CORE psychomotor retardation ratings.

**Table 1.** Demographic and clinical information

	cMDD (*n* = 47)	rMDD (*n* = 93)	Statistics
Age	29.01 (10.19)	28.7 (9.14)	*t*(138) = 0.18, *p* = 0.856, Cohen’s *d* = 0.02
Sex			X^2(1) = 2.2, *p* = 0.138
Female	74.5%	60.2%	
Male	25.5%	39.8%	
Race			X^2(3) = 1.59, *p* = 0.662
Asian	12.8%	11.8%	
Black or African American	19.1%	11.8%	
Multiracial	10.6%	14.0%	
White	57.4%	62.4%	
Ethnicity			X^2(1) = 0.66, *p* = 0.415
Hispanic/Latino	23.4%	16.1%	
Education (years)			X^2(6) = 2.36, *p* = 0.883
Grades 7 to 12	0.0%	1.1%	
High school or GED	2.1%	1.1%	
Partial college/Trade school	31.9%	25.8%	
2-year college or trade school	2.1%	3.2%	
4-year college	29.8%	38.7%	
Partial graduate school	17.0%	17.2%	
Graduate school	17%	12.90%	
Medication usage			
Antidepressants	34%	35.50%	X^2(1) = 0, *p* = 1
Anxiolytics	8.50%	2%	X^2(1) = 1.72, *p* = 0.189
Stimulants	2.10%	0%	X^2(1) = 0.12, *p* = 0.727
General depression (IDAS)	60.41 (11.92)	38.2 (10.02)	*t*(135) = 11.49, *p* < .001, Cohen’s *d* = 0.98
Psychomotor retardation (CORE)	2.07 (1.96)	1.93 (1.93)	*t*(98) = 0.33, *p* = 0.745, Cohen’s *d* = 0.03
Psychomotor agitation (CORE)	2.48 (1.99)	1.68 (1.61)	*t*(98) = 2.12, *p* = 0.037, Cohen’s *d* = 0.21

### Force variability task

**Figure 1.**
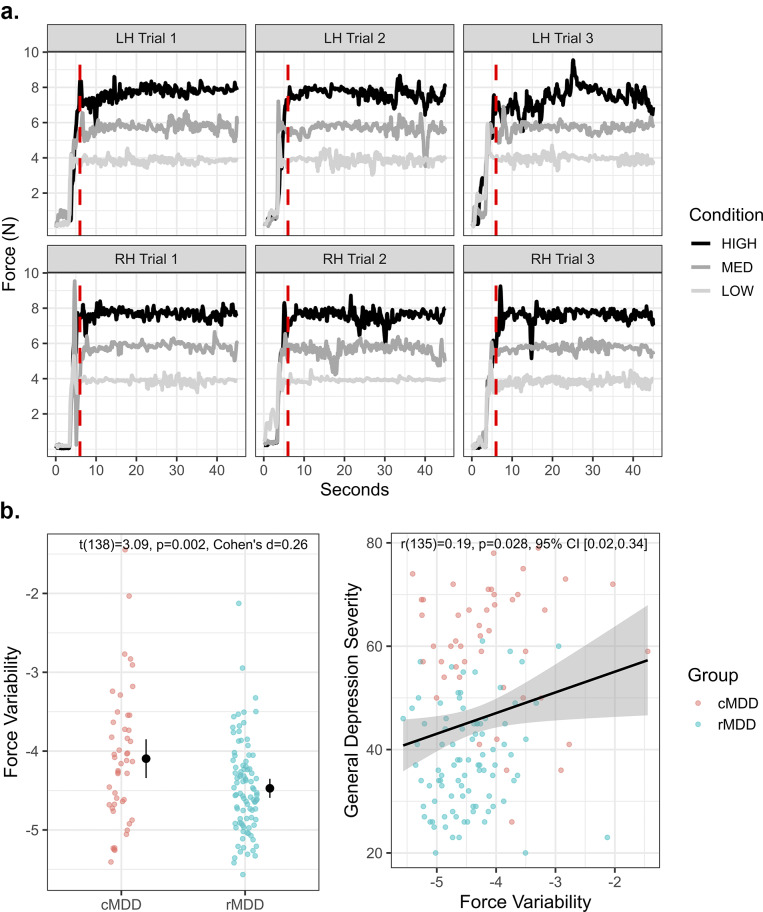
Force variability task and associations with depression. Panel (a) depicts all force variability task conditions for a single example subject. Data points to the left of the vertical red dotted lines (at 6 s) were removed. LH = Left hand; RH = Right hand. The left plot in panel (b) depicts group differences in force variability. The right plot depicts the association between force variability and general depressive symptoms as measured by the IDAS.

In the force variability task, participants used their index finger to apply consistent pressure to a force transducer (i.e. load cell). They were shown a real-time visual read out of the pressure exerted on the force transducer. They were also shown a “target” line indicating the amount of force they should aim to exert. There were three target force conditions: low (400 cN), medium (600 cN), and high (800 cN). Each force condition was completed three times with both left and right fingers such that there were 18 total trials (3 force conditions * 2 hand conditions * 3; see Figure 1a). Each trial was 45 s long, and the sampling rate of the transducer was 10 Hz.

During preprocessing, the first 60 samples (6 s) were removed from each trial to remove the initial force adjustment phase (see Figure 1). Force variability was measured as the coefficient of variation (CV), which was computed as the average force across a given trial divided by the standard deviation of the force for that trial. This operationalization of force variability is consistent with previous work in this area (Lohr et al., 2013; Lohr & Caligiuri, 2006; Mittal et al., 2013). Finally, the distribution of CVs was highly skewed, so we log-transformed the CV, which produced a roughly normal distribution.

### Resting state fMRI

MRI data were acquired using a 3 T Siemens MAGNETOM Prisma scanner. High-resolution anatomical images were collected with a T1-weighted MPRAGE sequence (FOV = 256 × 256 mm^2^; voxel size = 1 × 1 × 1 mm^3^; 178 slices; TR = 2170 ms; TI = 1190 ms; TE1 = 1.69 ms; TE2 = 3.55 ms; TE3 = 5.41 ms; flip angle = 7°). Resting-state functional images were acquired using a multiband-accelerated echo-planar imaging (EPI) sequence (FOV = 208 × 192 mm^2^; voxel size = 1 × 1 × 1 mm^3^; 64 slices; TR = 555 ms; TE = 22 ms; flip angle = 47°; multiband factor = 8). Head motion was monitored using the Framewise Integrated Real-Time MRI Monitoring (Dosenbach et al., 2017). Scanning continued until at least 10 min of usable data (defined as framewise displacement ≤0.2 mm) were obtained, with a maximum duration of 16 min. Participants were provided with feedback about their movement in-between scans. Groups did not differ in percentage of censored TRs (cMDD = 16.6%, rMDD = 15.0%, *t*(130) = 0.69, *p* = 0.491, Cohen’s *d* = 0.06).

Initial preprocessing steps were conducted for the anatomical and resting-state scans using fMRIPrep’s minimal preprocessing pipeline, version 20.2.0 (Esteban et al., 2019), a Nipype (v1.5.1)-based tool (K. Gorgolewski et al., 2011; K. J. Gorgolewski et al., 2017). Each T1w (T1-weighted) volume was corrected for intensity non-uniformity using N4BiasFieldCorrection v2.1.0 (Tustison et al., 2010) and skull-stripped using antsBrainExtraction.sh v2.1.0 (using the OASIS template). Brain surfaces were reconstructed using recon-all from FreeSurfer v6.0.1 (Dale, Fischl, & Sereno, 1999), and the brain mask estimated previously was refined with a custom variation of the method to reconcile ANTs-derived and FreeSurfer-derived segmentations of the cortical gray matter of Mindboggle (Klein et al., 2017). Spatial normalization to the ICBM 152 Nonlinear Asymmetrical template version 2009c (Fonov et al., 2011) was performed through nonlinear registration with the antsRegistration tool of ANTs v2.1.0 (Avants, Epstein, Grossman, & Gee, 2008), using brain-extracted versions of both T1w volume and template. Brain tissue segmentation of cerebrospinal fluid (CSF), white matter (WM) and gray matter (GM) was performed on the brain-extracted T1w using fast (Y. Zhang, Brady, & Smith, 2001). The BOLD EPI was skull-stripped and motion corrected (slice time correction was not done due to the MB accelerated acquisition). EPI was coregistered to a synthetic fieldmap and to the T1w structural image prior to registration to MNI 152 space.

Following preprocessing in fMRIprep, data were imported into the CONN toolbox version 21a (Whitfield-Gabrieli & Nieto-Castanon, 2012). Within CONN, functional data were smoothed using a default Gaussian kernel of 8 mm full width half maximum. The following nuisance regressors were entered into the first level GLMs based on Satterthwaite et al. (2013): white matter, cerebrospinal fluid, head motion (24 total motion parameters: x, y, z, pitch, roll, yaw; derivatives of these 6 motion parameters; squared terms of the 6 motion parameters and their derivatives), and motion outlier spike regressors (>.5 mm framewise displacement threshold). This motion outlier spike regressor was used to censor images with excessive motion. We did not include a global signal regressor (GSR) to avoid spurious negative correlations which were central to our hypotheses about dysconnectivity between brain regions (Murphy et al., 2009). However, when including GSR in the first-level models, primary results were unchanged. A default 0.008–0.09 Hz band-pass filter was applied to the time series after the first level GLMs.

### Connectivity analysis

Next, we computed ROI-to-ROI connectivity for each subject. Left- and right-hand knob ROIs were defined using a 10 mm sphere centered on the xyz coordinates [−38, −23, 56] and [38, −23, 56], respectively (Hardwick, Rottschy, Miall, & Eickhoff, 2013; Yousry et al., 1997). In brief, the hand knob is an area in the primary motor cortex (M1) that is contralaterally mapped to hand movements (Yousry et al., 1997). The ‘knob’ descriptor refers to the typical morphology of the area in which an Ω shape is formed by the precentral gyrus. Left and right early visual cortex ROIs were defined as the left and right intracalcarine cortex according to the FSL Harvard-Oxford Atlas maximum likelihood cortical atlas. We also used this atlas to define putamen and caudate ROIs. Finally, a cerebellar ROI (spanning lobules four and five) was defined according to the automated anatomical labeling (AAL) atlas (Tzourio-Mazoyer et al., 2002). Because we did not have any ROI laterality hypotheses, we averaged connectivity values across both hemispheres for all ROIs. Thus, a total of four ROI-to-ROI pairs were estimated: (1) motor hand area to visual cortex, (2) motor hand area to caudate, (3) motor hand area to putamen, and (4) motor hand area to cerebellum. Connectivity values were compared between groups using two-sample t-tests and correlated with symptom measures of depression using Pearson product moment correlations. We also ran mediation tests using the mediation package in R (Tingley et al., 2014). In short, we entered two regression models into the mediate() function: a linear ‘mediator model’ in which the independent variable (e.g. group status or depression severity) predicted the mediator (e.g. visuomotor connectivity), and a linear ‘outcome’ model in which the independent variable and mediator variable simultaneously predicted the outcome variable (e.g. force variability). For all of the mediation analyses in this manuscript, indirect effects were estimated via the product of coefficients approach. Standard errors were computed via the default quasi-Bayesian method with *n* = 5,000 simulations.

## Results

### Force variability task

Across all conditions of the force variability task, we observed greater force variability in cMDD relative to rMDD (see Figure 1b). We did not observe significant interactions of group by force condition (*F*(1.96,270.11) = 0.41, *p* = 0.662, η^2^ = .003), group by hand laterality (*F*(1,138) = 0.26, *p* = 0.613, η^2^ = .002) nor group by hand by force condition (*F*(1.92,264.53) = 2.78, *p* = 0.066, η^2^ = .020). Thus, there was little evidence of groups differing more for certain task conditions or hand laterality. Further details about task performance can be found in the Supplementary Material.

Greater force variability (averaged across conditions) was associated with increased clinician-rated psychomotor agitation (*r*(98) = 0.21, *p* = 0.032, 95% CI [0.02,0.39]), but not retardation (*r*(98) = −0.05, *p* = 0.653, 95% CI [−0.24,0.15]), as measured by the CORE. Greater force variability was also associated with more severe general depression symptoms (*r*(135) = 0.19, *p* = 0.028, 95% CI [0.02,0.34]). In exploratory follow-up correlations, we observed significant correlations with the following subscales of the IDAS: appetite loss (*r*(136) = 0.21, *p* = 0.016, 95% CI [0.04,0.36]), and Ill Temper (*r*(136) = 0.18, *p* = 0.038, 95% CI [0.01,0.33]).

### Cortico-subcortical connectivity

Force variability did not correlate significantly with connectivity between motor hand area and caudate (*r*(130) = −0.07, *p* = 0.445, 95% CI [−0.24,0.11]) or putamen (*r*(130) = −0.09, *p* = 0.322, 95% CI [−0.25,0.09]). Similarly, CORE psychomotor agitation ratings did not significantly correlate with connectivity between hand area and caudate (*r*(93) = −0.11, *p* = 0.275, 95% CI [−0.31,0.09]) or putamen (*r*(93) = −0.07, *p* = 0.511, 95% CI [−0.27,0.14]). However, cMDD did exhibit less connectivity between these regions than rMDD (caudate: *t*(130) = −2.55, *p* = 0.012, Cohen’s *d* = −0.22; putamen: *t*(130) = −2.24, *p* = 0.027, Cohen’s *d* = −0.19). General depression severity was associated with hand area to caudate connectivity (*r*(127) = −0.17, *p* = 0.049, 95% CI [−0.34,0]) but not with motor to putamen connectivity (*r*(127) = −0.12, *p* = 0.185, 95% CI [−0.28,0.06]). Thus, while cortico-striatal connectivity was sensitive to illness, it was not strongly associated with force variability.

We also examined connectivity between motor hand area and the cerebellum, specifically lobules 4 and 5 (Wüthrich et al., 2024). However, connectivity did not correlate with force variability (*r*(130) = 0.01, *p* = 0.894, 95% CI [−0.16,0.18]), CORE agitation clinical ratings (*r*(93) = −0.03, *p* = 0.761, 95% CI [−0.23,0.17]), depression severity(*r*(127) = 0.06, *p* = 0.475, 95% CI [−0.11,0.23]), and did not differ between groups (*t*(130) = 0.73, *p* = 0.468, Cohen’s *d* = 0.06).

### Visuomotor connectivity

**Figure 2.**
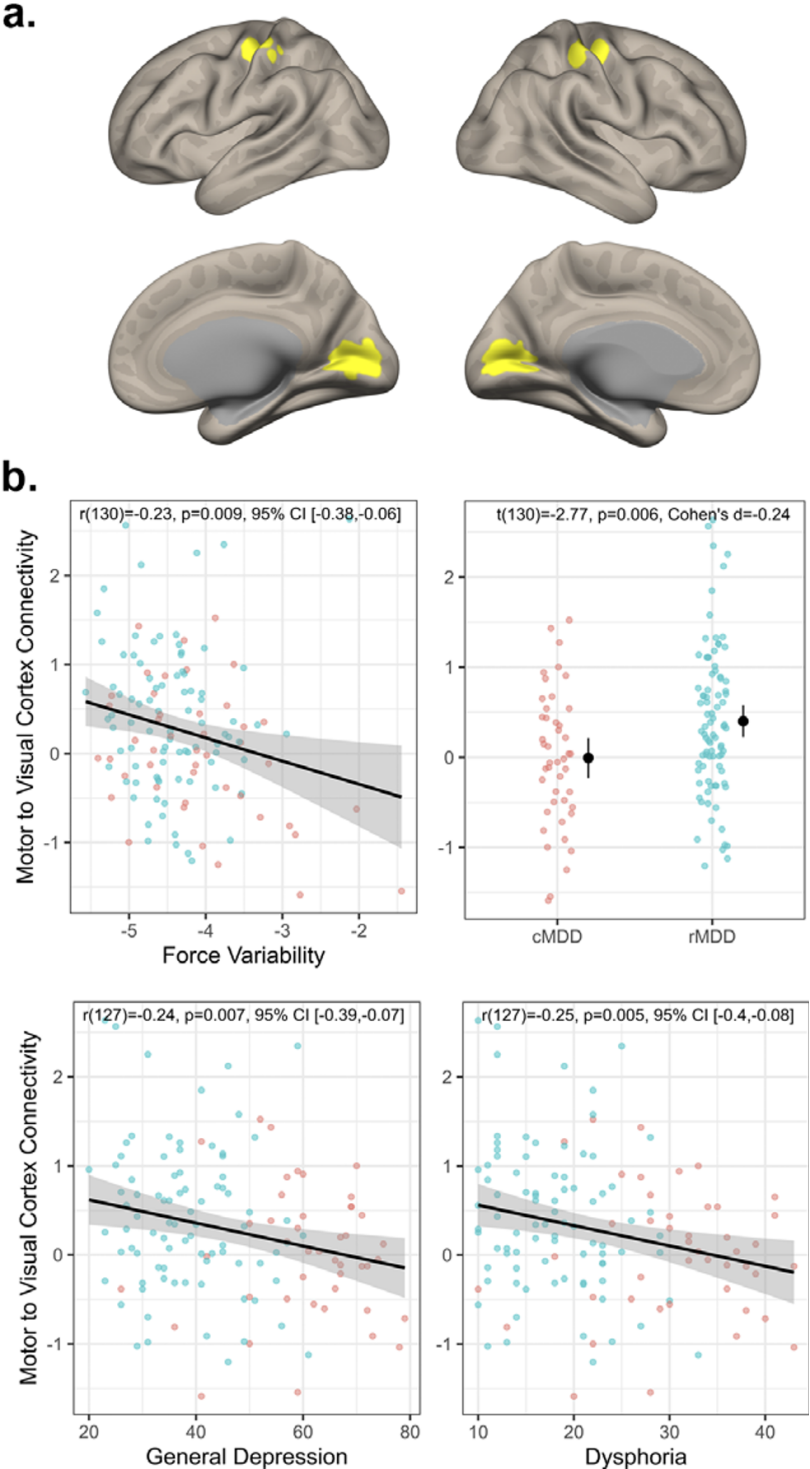
Visuomotor connectivity and associations with depression. Panel (a) depicts the motor and visual regions of interest. Panel (b) top left plot depicts the association between visuomotor connectivity values and force variability. Panel (b) top right depicts group differences in visuomotor connectivity. Panel (b) bottom row depicts associations between visuomotor connectivity, general depression, and dysphoria severity.

As depicted in Figure 2, across both groups, greater force variability was associated with less visuomotor connectivity (*r*(130) = −0.23, *p* = 0.009, 95% CI [−0.38, −0.06]). Additionally, rMDD individuals exhibited greater connectivity than cMDD (*t*(130) = −2.77, *p* = 0.006, Cohen’s *d* = −0.24). Connectivity values were also negatively associated with severity of general depression (*r*(127) = −0.24, *p* = 0.007, 95% CI [−0.39, −0.07]) and dysphoria (*r*(127) = −0.22, *p* = 0.011, 95% CI [−0.38, −0.05]) symptoms (see Figure 2b). In exploratory follow-up of more fine-grained IDAS scales, we again observed significant correlations with appetite loss (*r*(128) = −0.26, *p* = 0.003, 95% CI [−0.41, −0.09]) and Ill Temper scales (*r*(128) = −0.19, *p* = 0.029, 95% CI [−0.35, −0.02]). Finally, visuomotor connectivity values did not correlate with CORE agitation ratings (*r*(93) = −0.07, *p* = 0.507, 95% CI [−0.27, 0.13]).

**Figure 3.**
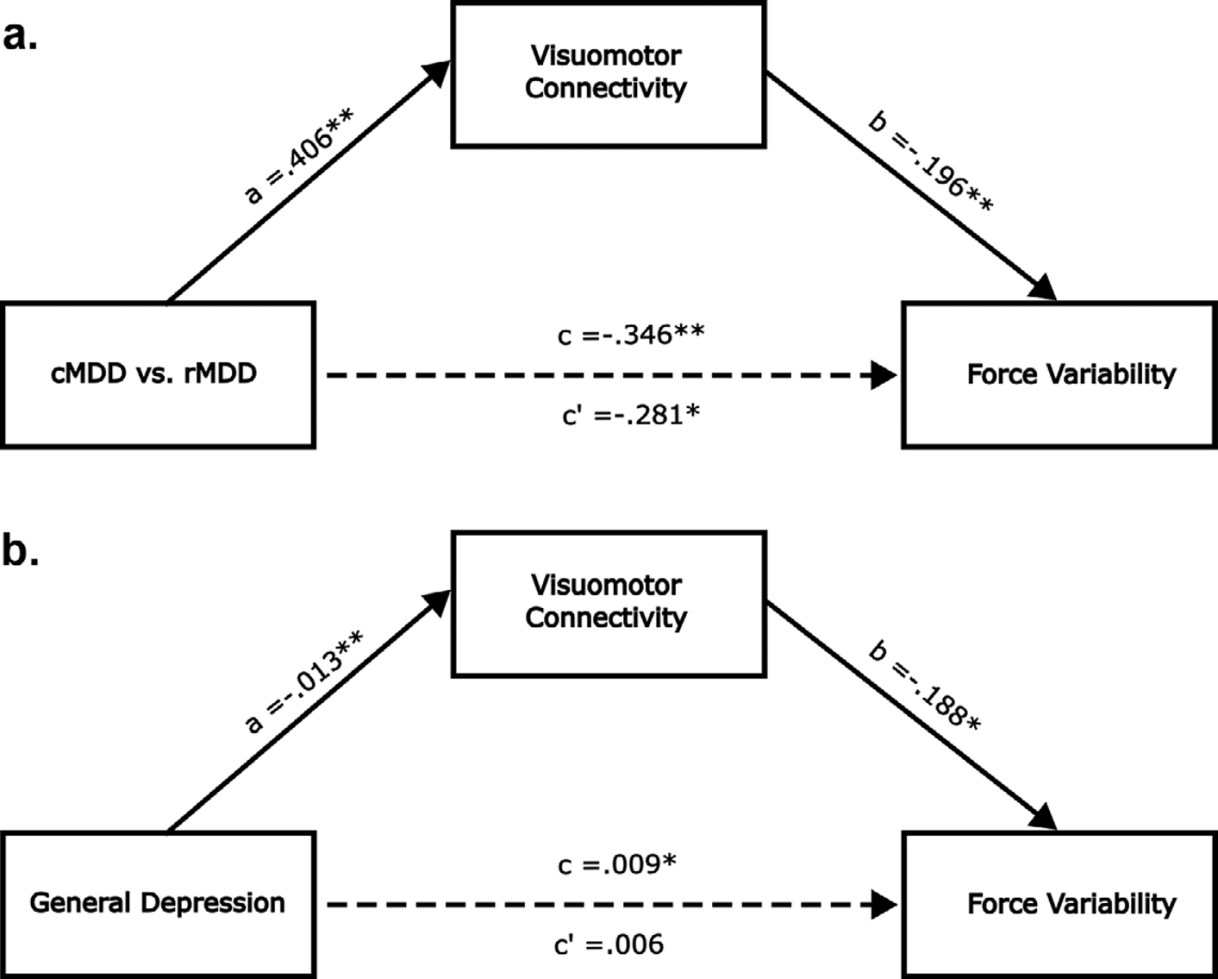
Visuomotor connectivity mediates association between depression and force variability. Panels (a,b) are identical except that the independent variable is group status in panel (a) and depression severity in panel (b). All path coefficients are unstandardized. c’ is the direct effect. c is the total effect. *** indicates *p* < .001, ** indicates *p* < .01 and * indicates *p* < .05.

Next, we tested whether visuomotor connectivity mediated the group difference in force variability. In this mediation model, group status was the independent variable, visuomotor connectivity was the mediating variable, and force variability was the dependent variable. As depicted in Figure 3a, visuomotor connectivity significantly mediated the group difference in force variability (ACME β = −0.06, 95% CI [−0.16, −0.001], *p* = 0.04). When entering general depression scores as the independent variable, we observed a similar mediating effect (ACME ACME β = 0.002, 95% CI [0.001, 0.005], *p* = 0.043). In this case, the direct effect between depression and force variability became insignificant (ADE β = 0.01, 95% CI [−0.002, 0.014], *p* = 0.146; see Figure 3b). We did not run mediation models with CORE ratings as the outcome due to the lack of association between the mediator variable (i.e. visuomotor connectivity) and the dependent variable (i.e. CORE agitation ratings). A meaningful association between mediator and outcome is generally considered a prerequisite for mediation analysis (Baron & Kenny, 1986). Similarly, we did not run models with cortico-striatal/cortico-cerebellar connectivity indices as the mediator variables due to the lack of association between these candidate mediator variables and the dependent variable (i.e. force variability).

## Discussion

In the present work, we identified altered visuomotor resting state functional connectivity as a candidate neural mechanism of instrumentally measured psychomotor agitation in MDD. We extended previous work by showing that force variability is (a) increased in cMDD relative to rMDD and (b) correlated with clinician-rated agitation scores. We then showed, for the first time, that visuomotor connectivity predicted force variability while, surprisingly, cortico-subcortical connectivity did not. Finally, we demonstrated that visuomotor connectivity is a plausible mediating mechanism of psychomotor agitation in depressed individuals.

### Force variability as specific instrumental measure of agitation

We are unaware of previous reports of a significant association between clinical ratings of psychomotor agitation and force variability. Lohr et al. (2013) examined force variability in a depressed sample with psychomotor disturbance but did not observe a significant correlation between force variability and psychomotor agitation. However, Lohr et al. (2013) used the HAM-D (Hamilton, 1960) to measure agitation, while we used the CORE. The CORE was specifically designed to capture psychomotor disturbance (Parker et al., 1993), while the HAM-D is a more general assessment of depression symptoms. Thus, it is possible that improved measurement of psychomotor agitation via the CORE can explain the discrepancy between the present findings and the findings of Lohr et al. (2013).

Force variability did not correlate with psychomotor slowing, suggesting that force variability may be a specific measure of psychomotor agitation rather than psychomotor disturbance more broadly. Thus, this instrumental measure appears to hold promise as a novel, valid, and objective measure of psychomotor agitation. Importantly, the correlation between force variability and agitation was relatively small (*r*(98) = 0.21), which is to be expected if the force variability measurement is, as hypothesized, capable of picking up on more subtle disturbances than traditional clinician ratings. This also may explain why visuomotor connectivity was significantly associated with force variability but not clinical ratings of agitation. However, measurement error and method-specific variance also likely attenuated the correlation between these measures. A critical next step of this work will be to assess the incremental validity of force variability, over-and-above clinician ratings, with respect to predicting outcomes of interest, such as treatment response, symptom trajectories, and real-world functioning.

### Visuomotor connectivity and implicated circuits

Motor cortex and subcortical motor areas like the basal ganglia and cerebellum have long been proposed as the primary neural mechanisms of psychomotor disturbance (Bracht et al., 2012, 2025; Lohr et al., 2013; Magioncalda et al., 2020; Martino et al., 2020; Walther et al., 2012; Wüthrich et al., 2024), with recent evidence implicating altered subcortical–cortical loops between SMN and the thalamus, substantia nigra, and raphe nuclei (Conio et al., 2020; Magioncalda et al., 2020; Martino et al., 2020). Still, others (Song et al., 2024) have argued for a broader account of psychomotor disturbance which implicates brain areas outside of the motor system. Consistent with this broader psychomotor account, Song et al. (2024) observed altered functional connectivity between motor cortex and visual cortex (specifically middle temporal visual complex) which related to clinician-rated psychomotor slowing. Our results provide convergent evidence of altered visuomotor connectivity in cMDD individuals, though our findings differ in a number of ways. While Song et al. (2024) observed altered connectivity between motor cortex and higher-level visual cortex (MT+), we focused on early visual cortex. Second, we focused on a sensitive instrumental measure of psychomotor *agitation* while Song et al. (2024) measured psychomotor *slowing* using clinician ratings. Still, despite these substantial differences, our studies converged on visuomotor connectivity as a candidate neural mechanism of psychomotor disturbance in MDD.

Our results also suggest that altered visual functioning is a critical, though understudied, feature of MDD. Several studies have observed reduced GABA concentrations in the visual cortex and altered visual surround suppression in MDD (Liu et al., 2022; Nickel et al., 2024; Salmela et al., 2021; Sanacora et al., 1999; Song et al., 2021). Importantly, Sanacora, Mason, Rothman, and Krystal (2002); Sanacora et al. (2003) observed increased GABA concentration in the occipital cortex of depressed individuals following both electroconvulsive therapy and selective serotonin reuptake inhibitor administration. More recently, Zhang et al. (2021) observed that repeated transcranial magnetic stimulation (rTMS) of the visual cortex led to decreased depressive symptom severity, accompanied by changes in resting state connectivity and functional activation of visual cortex. Together, these results highlight that alterations in the visual cortex are consistently associated with depression and that improvements in symptoms are often accompanied by changes in visual cortical functioning. This is consistent with the present findings in which remitted patients exhibited less visuomotor dysconnectivity than currently depressed individuals.

MDD severity is also thought to be related to dampening of visual perception (Fitzgerald, 2013), including perceived dimming of one’s ambient surroundings (Friberg, Bremer, & Dickinsen, 2008) and reduced color sensitivity (Barrick, Taylor, & Correa, 2002). While higher-level cognitive processes likely play a role, there is evidence that alterations in lower-level retinal functioning contribute to these perceptual blunting effects (Bubl et al., 2010). In particular, reduced contrast sensitivity in depression is argued to reflect altered dopaminergic neurotransmission in the retina, possibly reflecting a broader dopamine pathology throughout the brain (Bubl et al., 2009). Moreover, alterations in pupil light response in individuals with seasonal affective disorder (a type of major depression) are thought to reflect reduced light sensitivity, which may in turn disrupt circadian rhythms (Roecklein et al., 2013; Wescott et al., 2024). Future work should examine the degree to which such low-level visual dysfunction contributes to the visuomotor dysconnectivity observed in the present study.

Although we only observed significant associations between force variability and cortico-cortical visuomotor connectivity, it is possible, and perhaps likely, that subcortical circuits contribute to the altered visuomotor connectivity that we observed. Evidence for this perspective largely derives from animal studies in which monkeys are able to perform visually guided movements even after severing cortico-cortical white matter fibers connecting visual and motor areas (Glickstein, 2000; Myers, Sperry, & McCurdy, 1962). This suggests that subcortical pathways can support visually guided movements. In particular, Glickstein (Glickstein, 2000) argued for a cortico-ponto-cerebellar pathway in which visual information from the dorsal stream is passed to the cerebellum via the pontine nuclei. The cerebellum is then thought to communicate with the motor cortex via the ventral thalamus and basal ganglia (Bostan & Strick, 2018). Thus, while we observed altered functional connectivity between the early visual cortex and motor cortex, subcortical pathways may still be implicated.

In a recent review paper, Northoff et al. (2021) proposed three neurobiological mechanisms of psychomotor disturbance in psychiatric disorders. Of particular relevance to the findings of the present paper is the mechanism described as ‘modulation of motor cortex and motor network by non-motor cortical networks like default-mode network and sensory networks’. The evidence cited by Northoff et al. for this mechanism was primarily derived from studies of putative neuronal variability (measured via resting fMRI) in the sensorimotor, default mode, and visual sensory networks. Importantly, the activation of these networks seems to show reciprocal relationships with one another such that increased neuronal variability in the sensorimotor network (SMN) is accompanied by decreased variability in default mode and sensory networks. While our analyses quantified ROI-to-ROI connectivity, not neuronal variability, our results are consistent with disrupted modulation of visual and motor networks in depression that may contribute to psychomotor disturbance. Future work should explore whether the reduced visuomotor connectivity we report here is associated with alterations in the dynamic coordination of neuronal variability between SMN and sensory networks.

### Limitations

A limitation of the present study is that we did not collect functional MRI during force variability task performance (Dean et al., 2020). Thus, we cannot be sure that the connectivity patterns we observed at rest are reflective of those during task performance. Still there is a wealth of evidence demonstrating the substantial correspondence between resting and task-based functional connectivity (Cole et al., 2014; Cole, Ito, Bassett, & Schultz, 2016; Smith et al., 2009; Tavor et al., 2016). In particular, it has been postulated that resting connectivity can be thought of as a functional architecture through which neural activity flows during task performance (Cole et al., 2016). Still, future work should investigate the degree to which the resting connectivity patterns observed in the present manuscript correspond to connectivity patterns recorded during force variability task performance.

## Conclusion

The present work identified, for the first time, altered visuomotor connectivity as a candidate neural mechanism of psychomotor agitation in MDD individuals. Our results represent a critical step toward clarifying the pathophysiology of psychomotor agitation, an important indicator of clinical course and treatment responsiveness (Ulbricht et al., 2018). Furthermore, the present work demonstrates the utility and convergent validity of force variability as a laboratory measure of psychomotor agitation. Taken together, our findings point to the great potential of combining instrumental measures of psychomotor disturbance with functional neuroimaging to identify neural signatures, parse heterogeneity, and develop personalized treatments for depression.

## Supporting information

Pokorny et al. supplementary material Pokorny et al. supplementary material

## Supplementary material

The supplementary material for this article can be found at http://doi.org/10.1017/S0033291725102638.
