# Supplementary material for: Visuomotor dysconnectivity as a candidate mechanism of psychomotor agitation in major depression
Source: Psychol Med. 2025 Nov 28;55:e363. doi: 10.1017/S0033291725102638 (PMC12927545; doi:10.1017/S0033291725102638)
Supplement: Pokorny et al. supplementary material [file S0033291725102638sup001.docx]

**Supplemental Materials**

**Results**

*Extended Task Performance Results*

We also observed a smaller, but significant, effect of force condition (F(1.96,270.11) =3.25, p=0.041, η²=.023). This effect was driven by less force variability in the low force condition compared to the medium force condition. We also observed a force condition by hand laterality interaction (F(1.92,264.53)=32.04, p<.001, η²=.188) that was driven by greater force variability in the right hand compared to left for the medium force conditions but less force variability in the right hand compared to left for high force conditions.

**Discussion**

*Associations with Ill Temper*

An unexpected, but intuitive, finding is that both force variability and visuomotor connectivity were correlated with the Ill Temper scale of the IDAS. Ill temper, specifically irritability, is often considered to be an indicator of psychomotor agitation. Thus, the association between force variability and ill temper provides encouraging convergent evidence for force variability as a valid measure of psychomotor agitation. Furthermore, this pattern of results may be useful for parsing heterogeneity in depression. In particular, it is theorized that irritability may be an indicator of anxious depression, a subtype of depression. For example, Ayvaci et al. [(2024)](https://paperpile.com/c/O2PZai/30T1A/?noauthor=1) observed that youth with anxious depression reported higher rates of irritability and psychomotor agitation, as compared to youth without anxious depression. This profile aligns closely with the observed correlations in the present manuscript. Still the causal nature of these associations are unclear: irritability may cause increased force variability or irritability and force variability may be caused by a third variable (e.g., negative affect). Experimentally manipulating irritability through a frustrating force variability task would be one way of disentangling the causal directionality of this association: if heightened irritability causes psychomotor agitation, then force variability should be increased for frustrating task conditions (assuming frustration provides a reasonable proxy for irritability).

*Lack of Group Difference in Psychomotor Slowing*

In the current study, cMDD was associated with higher levels of psychomotor agitation than rMDD but psychomotor slowing did not differ between groups. It is possible that this reflects a scarring effect specific to psychomotor slowing in which, even after other mood symptoms have subsided, psychomotor slowing does not. This is consistent with the findings of a recent meta-analysis in which actigraphically measured psychomotor slowing was evident in both current and remitted depressed individuals [(Wüthrich et al., 2022)](https://paperpile.com/c/O2PZai/ag7lm). Still we are careful to note that absence of evidence for a group difference is not evidence of absence [(Altman & Bland, 1995)](https://paperpile.com/c/O2PZai/ehV6Y). Additionally, other studies of psychomotor disturbance have focused on more chronic, severe and treatment resistant samples [(Baeken et al., 2010; Ulbricht et al., 2018; van Diermen et al., 2019)](https://paperpile.com/c/O2PZai/dGZKD+wKA0I+uRBKb), while our sample consisted of outpatients such that the range of psychomotor disturbance may have been restricted. Having said this, the CORE scores we observed were roughly equivalent to scores reported in previous work [(Attu et al., 2012)](https://paperpile.com/c/O2PZai/A2He9). Thus, the lack of group difference does not seem to be driven by a general lack of psychomotor slowing in our sample.
